# The global economic burden of health anxiety/hypochondriasis- a systematic review

**DOI:** 10.1186/s12889-023-17159-5

**Published:** 2023-11-13

**Authors:** Kawka Hannah, Kurtz Marie, Horstick Olaf, Brenner Stephan, Deckert Andreas, Lowery Wilson Michael, Baernighausen Till, Dambach Peter

**Affiliations:** Heidelberg Institute for Global Health (HIGH), Faculty of Medicine and University Hospital, Heidelberg University, 69120 Heidelberg, Germany

**Keywords:** Economic Burden, Hypochondriasis, Health anxiety, Illness anxiety, Costs

## Abstract

**Background:**

Recent studies have shown a lifetime prevalence of 5.7% for health anxiety/hypochondriasis resulting in increased healthcare service utilisation and disability as consequences. To the best of our knowledge, there has been no systematic review examining the global costs of hypochondriasis, encompassing both direct and indirect costs. Our objective was to synthesize the available evidence on the economic burden of health anxiety and hypochondriasis to identify research gaps and provide guidance and insights for policymakers and future research.

**Methods:**

A systematic literature search was conducted using PubMed, Web of Science, PsycInfo, EconLit, IBSS and Google Scholar without any time limit, up until April 2022. The Preferred Reporting Items for Systematic Reviews and Meta-Analyses (PRISMA) guidelines were followed in this search and the following article selection process. The included studies were systematically analysed and summarized using a predefined data extraction sheet.

**Results:**

Of the 3044 articles identified; 10 publications met our inclusion criteria. The results displayed significant variance in the overall costs listed among the studies. The reported economic burden of hypochondriasis ranged from 857.19 to 21137.55 US$ per capita per year. Most of the investigated costs were direct costs, whereas the assessment of indirect costs was strongly underrepresented.

**Conclusion:**

This systematic review suggests that existing studies underestimate the costs of hypochondriasis due to missing information on indirect costs. Furthermore, there is no uniform data collection of the costs and definition of the disease, so that the few existing data are not comparable and difficult to evaluate. There is a need for standardised data collection and definition of hypochondriasis in future studies to identify major cost drivers as potential target point for interventions.

## Introduction

Hypochondriasis or, sometimes also referred to as health anxiety, can be broadly defined as the pathological fear of suffering from a serious physical illness, poses a major economic burden on healthcare systems and society. One of the most recent studies on the prevalence of hypochondriasis in the general population was conducted in Australia, and found a lifetime prevalence of 5.7%, with a peak in middle age [1]. A systematic review on the prevalence of hypochondriasis by Weck et al. showed a range of 0.3–8.5% in a general medical setting (e.g., primary care) [2]. Data on the prevalence of hypochondriasis in children are scarce. In the Copenhagen Child Cohort Study, parents were asked about health anxiety symptoms in their children aged between 5 and 7 years. The study revealed that 17.6% of the cohort had symptoms, and 2.4% had significant symptoms [3]. The condition is associated with an increased use of health services due to regular doctor visits and elevated number of days on sick leave, compared to the general population, as patients suffering from hypochondriasis are convinced or afraid that they might have a serious illness [1, 4, 5].

There seems to be no uniform definition for health anxiety and hypochondriasis. In the literature, these two terms are often used interchangeably, but some authors define them as two separate entities. The DSM IV (Diagnostic and Statistical Manual of Mental Disorders) categorizes hypochondriasis as a somatoform disorder involving the misinterpretation of physical sensations [5]. However, this definition has been criticized by several authors, who have called for reclassification. For example, Olantunji et al. argue that the focus of the disorder is on illness anxiety rather than somatic complaints [6]. In response to the criticism, hypochondriasis was replaced in the DSM V by two new diagnoses: somatic symptom disorder and illness anxiety disorder [7].

In terms of therapy, cognitive behavioural therapy (CBT) is considered the most effective treatment for the disease and can also be offered as an online therapy [8]. It is assumed that digitalization and almost unlimited access to information via the internet further fosters increases in the prevalence of health anxiety/hypochondriasis, which, in turn could lead to increased healthcare costs [9]. Excessive online searches for symptoms of illness are also referred to as cyberchondria [10].

Health costs can be categorized into different types. There are the tangible, monetarily measurable costs. They differ in the cost perspective, i.e., whether they are incurred by the service provider, the patient, or society. Tangible costs are further classified into direct and indirect costs. Direct costs are expenses directly related to the disease, such as transport costs, expenditures for specialized medication, home modifications due to a disease, or therapies that the patient has to pay for out of pocket. If therapy or prevention is covered by health insurance, these direct costs are incurred by the service providers. Administrative costs, personnel costs, expenses for doctor’s visits and diagnostics, or research are also considered direct costs. Indirect costs are reflected in productivity losses, increased absenteeism from work due to sick leave and the associated loss of wages. Another type of costs are intangible costs, which will not be discussed in detail here [11].

To the best of our knowledge, no existing systematic review has examined the economic impact of health anxiety and hypochondriasis on healthcare systems and societies at a global level. This paper aims to synthesize the available evidence on the economic burden of health anxiety and hypochondriasis, specifically focusing on total costs, including both direct costs of health anxiety/hypochondriasis caused by doctor visits, medication or administration and indirect costs resulting from absenteeism from work and productivity loss.

As the first systematic review in this field, a key objective is to emphasize the disease burden, enabling resource allocation, healthcare prioritization, and identifying research gaps to provide guidance and insights for policymakers and future research.

## Methods

This systematic review was conducted using the Preferred Reporting Items for Systematic Reviews and Meta-Analyses (PRISMA) [12]. It is registered on the PROSPERO register for systematic reviews (registration number: CRD42021240018).

### Search strategy

We started a preliminary search to detect relevant search terms and develop a search string consisting of (i) the disease hypochondriasis and related synonyms and (ii) search terms relating to economic burden. The latter included the terms „health anxiety“, „hypochondria*“, and „illness anxiety disorder“. The second part included the terms „cost*“ and „economic“. An asterisk has been added to the terms cost and hypochondria to include other terms with the same root word in the search. Search terms within a category were linked with the Boolean operator „OR“ and then enclosed with brackets, the categories themselves where then linked with „AND“.

We conducted the systematic literature search in the following databases: PubMed, Web of Science, PsycInfo, EconLit, IBSS and Google Scholar. No restriction on publication year was applied. In Google Scholar, we screened the results for each search combination to saturation which means we continued screening until no more relevant articles appeared, and our subject area was adequately covered. After reaching saturation, 30 more articles were screened. Saturation was achieved within a range of 50 to 180 articles depending on the search term used. Later, we screened the reference lists of the included publications to detect further relevant articles. The search was conducted in April 2022.

### Study selection

All database hits were imported into Rayyan to remove duplicates and to screen the records [13]. The title and abstract screening against eligibility criteria was carried out independently by HK and MK. The manuscripts whose title and abstract met the inclusion criteria underwent full-text screening. Disagreements and uncertainties were clarified by either bilateral consensus or discussion among all authors.

### Eligibility criteria

We included any peer-reviewed study on the economic burden of hypochondriasis, such as direct costs, related doctor visits both primary and secondary care, medication, or administration as well as indirect costs resulting from absenteeism or productivity losses. Our systematic review included studies from low-, middle- and high-income countries. We encompassed all types of studies, including randomised controlled trials (RCT), quasi-experimental studies, observational studies, cohort studies, cross-sectional studies, cost-effectiveness, cost-utility, and cost-of-illness.

We only included articles that reported costs incurred by health anxiety/hypochondriasis if the patients did not receive any special therapy like cognitive behavioural therapy. The rationale behind this criterion is that hypochondria still receives limited attention in diagnosis and treatment. Therefore, we aimed to showcase the costs that arise, particularly from untreated cases, to underscore the importance of adequate diagnosis and subsequent therapy for the disease. For comparative studies examining the cost of different forms of cognitive behavioural therapy, only the costs incurred by the control group, such as a waiting list control or treatment as usual (TAU), were included.

There was neither an age limitation of participants nor a date restriction regarding the publication date. Consequently, the included population consists of adults, young adults, and children. Included publications had to be indexed in English language.

### Exclusion criteria

We did not consider studies that analysed the cost of cognitive behavioural therapy (CBT) as well as internet-guided CBT (iCBT) as treatment for health anxiety. For better comparability and to examine the total costs objectively, we excluded studies that did not provide monetary values of incurred cost items, such as the frequency of doctor visits or number of days absent from work. Due to low quality of evidence, we did not include case reports, narrative reviews, expert opinions, or editorials.

### Data extraction and cost conversion

The included studies were subjected to systematic analysis, and the relevant information was summarized in a predefined data extraction sheet. This data extraction sheet includes general information such as the title, author, journal, date of publication, country, study type, study objective, study setting, source of data, diagnostic assessment tool, definition of the disease used, and the time frame of the study. Additionally, the characteristics of the study participants, such as the number of participants, gender, and age, were analysed. Regarding our primary outcome, the total costs, as well as the different types of costs, were collected along with the corresponding currency. As reported costs related to different years, currencies and countries, all cost information was converted to US$ for the year 2022 using the CCEMG - EPPI-Centre Cost Converter [14].

### Quality assessment

We assessed the quality of included studies using the reporting guidelines suggested by the equator network [15]. In the case of observational studies or RCTs, we used the STROBE (Strengthening the Reporting of Observational Studies in Epidemiology) and CONSORT (Consolidated Standards of Reporting Trials) checklists for the assessment. The basis of the evaluation was the number of fulfilled characteristics listed in the checklist. We scored one point if the criterion was completely fulfilled, half a point if it was partially fulfilled and no point if the item was not mentioned in the article. A reporting less than 70% was defined as low reporting quality, while 85% and above was classified as high quality. Everything in between was defined as medium quality. The quality assessment was also carried out independently by HK and MK. Disagreements were solved by discussion until consensus was reached. No studies were excluded due to poor reporting.

## Results

### Study selection

In total, our database search in 2022 yielded 3044 articles. After the duplicates were removed, 19 articles remained for full-text screening. The screening of references of included articles did not reveal any further studies.

**Fig. 1:**
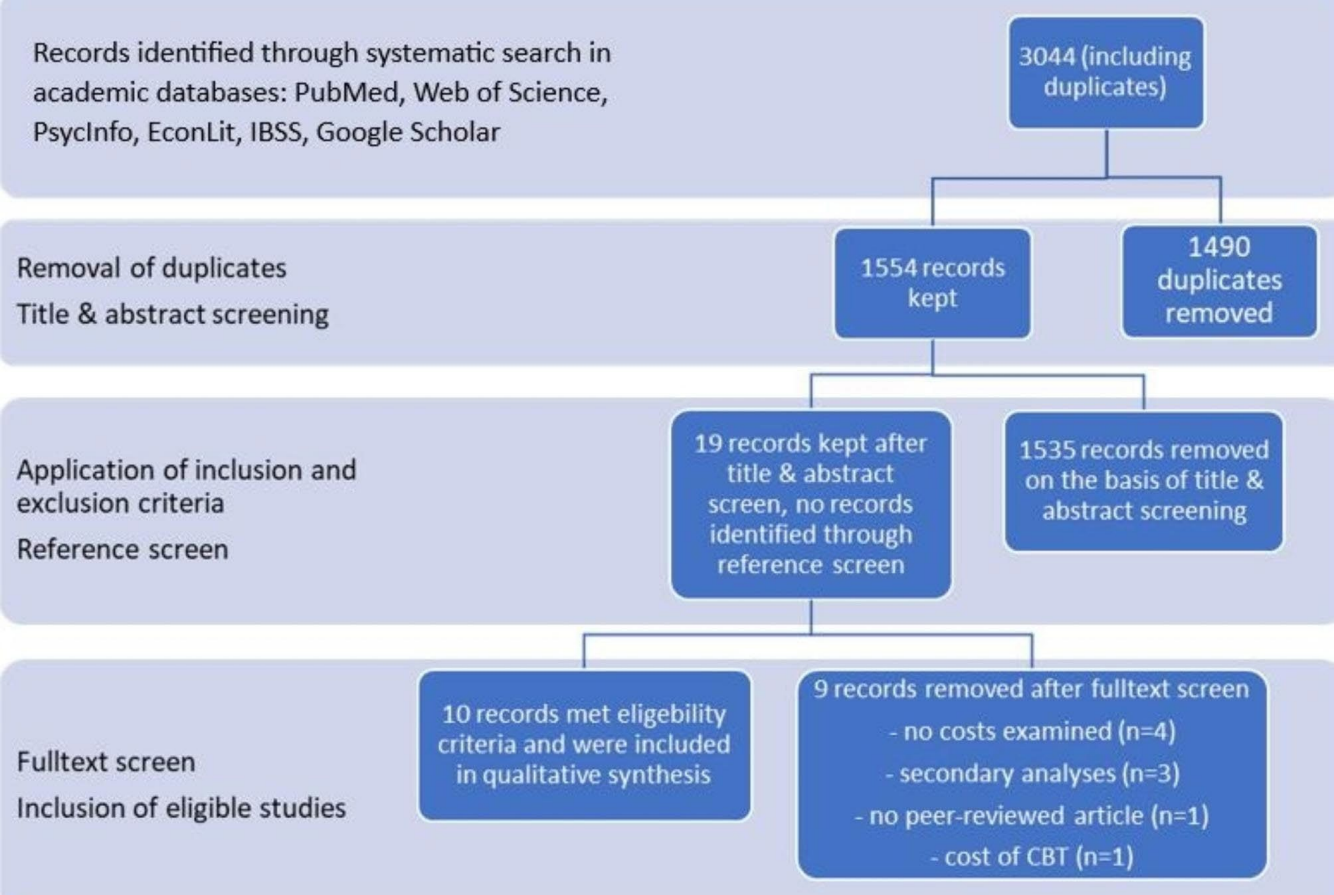
Flow diagram of literature search and study selection

Since three articles represented secondary analyses of already included studies, data of those studies were no longer considered. Four studies were excluded as there were no costs examined and one study was not peer-reviewed. Another study only looked at the costs of CBT without a control group. Finally, 10 studies met the inclusion criteria and were included in the systematic review. (Fig. 1).

**Table 1:** Summary of study characteristics

Authors	Publication Year	Country	Study Setting	Sample Size (n) Subgroups	Sex Female, n (%)	Age (years) i.) Age limit for inclusion ii.) Study population	Time Frame a) Data Collection b) Follow-up	Tool and Quality of Reporting
**Non-interventional studies for health anxiety/hypochondriasis**								
Barsky A. et al. [18]	2001	USA	Primary care clinic of Brigham and Women’s Hospital, Boston	876 $\ge$ST: 212 $<$ST: 664	$\ge$ST: / (75) $<$ST: / (72)	i.)$\ge$18 ii.) /	a) 1997-1998 b) 12 months	STROBE 68%
Fink P. et al. [19]	2010	Denmark	Primary care patient’s consulting their family Physicians	1129 mild HA: 21 severe HA: 81 DSM-IV: 59 CC: 968	mild HA: / (61.9) severe HA: / (71.6) DSM-IV: / (79.6) CC: / (58.9)	i.) 18-65 ii.) mean (SD) mild HA: 39.1 (13.1) severe HA: 39.7 (13.7) DSM-IV: 38.4 (11.3) CC: 37.8 (13.1)	a) 2004 b) 3, 12, and 24 months	STROBE 79%
**Control groups of cognitive behaviour therapy (CBT) studies for health anxiety/hypochondriasis**								
Tyrer P. et al. [26]	2014	United Kingdom	Six general hospitals covering urban, suburban, and rural areas	444 CBT: 219 CC: 225	CBT: 113 (52) CC: 123 (55)	i.) 16-75 ii.) mean (SD) CBT: 50.3 (13.6) CC: 47.0 (13.4)	a) 2008-2010 b) 24 months	CONSORT 78%
Morriss R. et al. [21]	2019	United Kingdom	Primary and secondary care centres across the East Midlands and at two other English sites	156 RCBT: 78 CC: 78	RCBT: 56 (72) CC: 52 (67)	i.)$\ge$18 ii.) median (range) RCBT: 31.12 (18.36-70.10) CC: 33.28 (18.69-82.71)	a) 2014-2016 b) 12 months	CONSORT 78%
Seivewright H. et al. [25]	2008	United Kingdom	Genitourinary medicine clinic King’s Mill Hospital, Sutton-in-Ashfield	49 CBT: 23 CC: 26	23 (/) of total sample size	i.) 16-65 ii.) /	a) 2002-2005 b) 12 months	CONSORT 74%
Hedman E. et al. [20]	2012	Sweden	Karolinska University Hospital, Stockholm	81 iCBT: 40 CC: 41	iCBT: 28 (/) CC: 32 (/)	i.) / ii.) mean (SD) iCBT: 39.3 (9.8) CC: 38.8 (9.5)	a) / b) 12 months	CONSORT 66%
Risør et al. [24]	2022	Denmark	Research Clinic for Functional Disorders and Psychosomatics at Aarhus University Hospital	101 iACT: 53 iFORUM: 48	iACT: 34 (64) iFORUM: 32 (67)	i)$\ge$18 ii) mean (SD) iACT: 37.2 (9.7) iFORUM: 42.3 (9.6)	a) 2016-2017 b) 6 months	CONSORT 68%
Axelsson et al. [17]	2018	Sweden	Karolinska University Hospital, Stockholm	132 G-ICBT: 32 U-ICBT: 33 BIB-CBT: 34 WLC: 33	G-ICBT: 24 (75) U-ICBT: 24 (73) BIB-CBT: 24 (71) WLC: 36 (79)	i)$\ge$18 ii) mean (SD) G-ICBT: 38.6 (12.6) U-ICBT: 37.4 (11.6) BIB-CBT: 35.4 (12.4) WLC: 41.5 (13.5)	a) 2013-2016 b) 12 months	CONSORT 73%
**Health anxiety in children**								
Rask C. U. et al. [22]	2015	Denmark	Copenhagen Child Cohort 2000	1884 low HA: 184 intermediate HA: 1539 high HA: 161	low HA: 76 (41.3) intermediate HA: 812 (52.8) high HA: 99 (61.5)	i.) 11-12 ii.) /	a) 2011-2012 b) /	STROBE 81%
Rimvall M. K. et al. [23]	2020	Denmark	Copenhagen Child Cohort 2000	1884 (2011) 1278 (2016)	707 (55.3) of total sample size 2016	i.) 11 and 16 ii.) /	a) 2011-2012; 2016-2017 b) /	STROBE 78%

≥ST above or equal Somatization Threshold; <ST below Somatization Threshold; ≥ 18 older than or equal to; / not reported; STROBE The Strengthening the Reporting of Observational Studies in Epidemiology, CONSORT Consolidated Standards of Reporting Trials; HA Health anxiety; DSM-IV Hypochondriasis Diagnosis according to Diagnostic and Statistical Manual; CC Control Condition; SD Standard Deviation; CBT Cognitive Behaviour Therapy; RCBT Remotely delivered Cognitive Behaviour Therapy; ICBT Internet Based Cognitive Behaviour Therapy; iACT Internet-Delivered Acceptance and Commitment Therapy; iFORUM Internet Forum; G-ICBT Therapist-Guided Internet Cognitive Behavioural Therapy; U-ICBT Unguided Internet Cognitive Behavioural Therapy; BIB-CBT Cognitive Behavioural Bibliotherapy; WLC Waiting List Control.

**Table 2:** Reported total costs and adjusted costs for the financial year 2022 of health anxiety/hypochondriasis

Country	Source of cost information	Diagnostic assessment tool	Type of costs	Period of measurement	Total cost per patient per year	Currency	Total annual per capita costs converted to US$ adjusted for the financial year 2022
**USA** [18]	Hospital administrative and accounting databases	Whiteley Index	**Direct outpatient cost**: 1) physician contacts (primary and specialized care). 2) labs and procedures.	a) preceding 12 months to index visit b) following 12 months	a)$$\ge$$ST: 1312.00* b)$$\ge$$ST: 1395.00*	US dollars (financial year not reported)	a)$$\ge$$ST: 1964.91 b)$$\ge$$ST: 2089.21
**Denmark** [19]	National Health Service Register, National Patient Register, National Psychiatric Central Register, National Board of Health	Schedules for Clinical Assessment in Neuropsychiatry (SCAN)	**Direct outpatient cost**: 1) primary and specialized care 2) medicines **Other direct costs**: 3) general hospital care (including emergency room care) 4) psychiatric care	a) preceding 36 months to index visit b) following 24 months	mild HA: a) 512.00 b) 672.00 severe HA: a) 1766.00 b) 1533.00 DSM-IV: a) 1200.00 b) 1099.00	Euros adjusted for the financial year 2004	mild HA: a) 857.19 b) 1125.06 severe HA: a) 2956.62 b) 2566.54 DSM-IV: a) 2009.03 b) 1839.94
**United Kingdom** [26]	Hospital records	Health Anxiety Inventory (HAI) Structured Clinical Interview for DSM-IV hypochondriasis	**Direct outpatient cost**: 1) general practitioner contacts, 2) medication 3) community health and social care contacts **Other direct costs**: 4) hospital services, 5) inpatient accommodation	24 months	3863.70	GBP adjusted for the financial year 2008-09	7088.98
**United Kingdom** [21]	National Health Service	Short Health Anxiety Inventory (SHAI)	**Direct outpatient cost**: 1) hospital visits, 2) primary and community care **Other direct cost**: 3) hospital visits 4) medication 5) travel 6) informal care	12 months	3261.00	GBP adjusted for the financial year 2017	5161.18
**United Kingdom** [25]	National Health Service	Short Health Anxiety Inventory (SHAI)	**Direct cost for primary and secondary health service use**: 1) primary care contacts 2) out-patient appointments 3) in-patient stays 4) A&E attendances	12 months	634.00	GBP adjusted for the financial year 2004-05	1294.95
**Sweden** [20]	Official health-care tariffs	Health Anxiety Interview Health Anxiety Inventory (HAI) The Illness Attitude Scale Whiteley Index	**Direct outpatient cost**: 1) healthcare visits 2) medications **Other direct costs**: 3) non-medical **Indirect costs**: 4) unemployment 5) sick leave 6) work cutback 7) domestic productivity	12 months	8536.00	GBP adjusted for the financial year 2010	15179.29
**Denmark** [24]	National Health Service Register	Whiteley Index Short Health Anxiety Inventory (SHAI)	**Direct outpatient costs**: 1) general practitioners 2) psychiatrists 3) medical specialists 4) psychologist 5) therapists 6) other **Secondary care** 7) somatic hospital inpatient 8) somatic hospital outpatient 9) psychiatric hospital outpatient **Indirect costs** 10) productivity loss (sick leave)	a) baseline b) 6 months	a) 1700.00 b) 1289.00	Euro adjusted for the financial year 2018	a) 2366.83 b) 1794.61
**Sweden** [17]	Official health-care tariffs, market prices, human capital approach	Mini international neuropsychiatric interview	**Direct outpatient costs** 1) healthcare visits 2) medication **Other direct costs** 3) non-medical **Indirect Costs** 4) unemployment 5) sick leave 6) work cutback 7) domestic	a) baseline b) post-treatment	a) 12768.00 b) 7872.00	GBP adjusted for the financial year 2014	a) 21170.71 b) 13052.62
**Denmark** [22]	National Health Service Register	Childhood Illness Attitude Scale	**Direct outpatient cost** 1) primary and specialized care contacts 2) lab tests 3) x-rays	24 months	Low HA: 80.96 Intermediate HA: 115.18 High HA: 154.99	Euro adjusted for the financial year 2014	Low HA: 120.52 Intermediate HA: 171.47 High HA: 230.73
**Denmark** [23]	National Health Service Register	Childhood Illness Attitude Scale	**Direct outpatient cost** 1) primary and specialized care contacts 2) lab tests 3) x-rays	12 months	Low HA 2011/2012: 124.00 2015/2016: 115.00 High HA 2011/2012: 246.00 2015/2016: 191.00	Euro adjusted for the financial year 2016	Low HA 2011/2012: 177.98 2015/2016: 165.06 High HA 2011/2012: 353.09 2015/2016: 274.15

$\ge$ST above or equal Somatization Threshold; DSM-IV Hypochondriasis Diagnosis according to Diagnostic and Statistical Manual; HA Health anxiety; GP General Practitioner; GBP Great Britain Pound; A&E Attendances and Emergency Admissions; *outcome measure adjusted for patient’s sex, age group, type of insurance, and medical comorbidity

### Results of general study characteristics

Geographically, the ten studies were conducted in four different countries (United States, Sweden, Great Britain/United Kingdom, Denmark), which all can be classified as high-income countries according to the World Bank income group classification [16]. Altogether they were covering only two regions: Europe and North America [17–26].

Two studies were non-interventional studies that compared the costs caused by hypochondriasis with the costs incurred by participants with other well-defined medical conditions [18, 19]. Six studies examined the cost-effectiveness of CBT, iCBT or internet delivered Acceptance and Commitment Therapy but included control groups that could be used to extract data for this systematic review [17, 20, 21, 24–26]. In four studies, the control groups received standard care. Participants assigned to standard care did not receive specific therapy but had the option to present to their general practitioner or clinic if needed. In two of these studies, it was explicitly mentioned that the patients were informed about the disease during the recruitment process, and in one study, their general practitioner was also informed about it [17, 21, 25, 26]. In two studies, the control group was given access to an online forum for peer interaction with other participants [20, 24].

While eight of the studies focused on adult participants suffering from health anxiety/hypochondriasis [17–21, 24–26], two studies examined health anxiety in children. These studies are part of the Copenhagen Child Cohort 2000 and were conducted by the same research team [22, 23]. The more recent of the two studies was a follow-up study of the previous one [23].

All studies contained information on the sex distribution of the study participants, either in the form of percentages, absolute values, or both [17–26]. One study did not provide information on the sex distribution with respect to the two study arms [25]. All studies were published between 2001 and 2022 [17–26]. Only six studies provided summarized information on the age distribution of study participants [17, 19–21, 24, 26] while Barsky et al. provided a frequency distribution for different age groups [18]. Since the data from the Copenhagen Child Cohort 2000 were collected from the same participants in 2011–2012 and again in 2016–2017, the children were initially 11–12 and at follow-up 16–17 years old [22, 23]. The cost assessment conducted by Rask et al. referred to the 2 years prior to data collection [22].

For detailed information see Table 1. In terms of quality assessment using the STROBE and CONSORT checklist, 7 studies showed medium reporting quality [17, 19, 21–23, 25, 26], while three studies showed low quality of reporting [18, 20, 24].

### Results of definition of hypochondriasis/health anxiety

Most of the included studies used the terms health anxiety and hypochondriasis interchangeably and employed similar assessment tools to identify the study participants [20, 21, 24–26]. Fink et al. replaced hypochondriasis with health anxiety due to stigmatization and introduced a classification system distinguishing between mild and severe health anxiety [19]. The aim of this new classification was to address the overlap of DSM-IV hypochondriasis with other somatoform disorders [19, 27].

The earliest study examined both hypochondriacal health anxiety and somatisation. The patients were given a questionnaire inquiring about somatic symptoms and hypochondriacal fears. Based on a predefined cut-off, the patients were classified as being above or below the somatisation threshold (see Table 1). Thus, an intersection of these two syndromes is examined here. Since at that time the scientific world made less of a distinction between hypochondriasis and somatisation disorders and, according to Barsky et al., all patients showed clinically relevant levels of hypochondriasis, we decided to include this publication nevertheless [18].

Only one study used the new DSM-V definition, which distinguishes between somatic symptom disorder and illness anxiety disorder. Both entities were accepted as eligibility criteria for recruitment [17]. In the children’s studies, the term health anxiety symptoms were used as there is still no clear definition of the disease in childhood [22, 23, 28].

### Reported total costs and data source

Information extracted on the costs of hypochondriasis are shown in Table 2. Cost information was collected from administrative hospital databases (n = 1), hospital records (n = 1), questionnaires and official cost tariffs (n = 2) and national health registers (n = 6) [17–26]. While all studies reported direct medical costs related to outpatient care, these do not consistently represent identical cost components across studies, as can be seen in the “Type of Costs” column of Table 2. Additional direct medical and non-medical costs were reported by five studies [17, 19–21, 26], while three studies also estimated indirect costs related to hypochondriasis [17, 20, 24].

### Total costs of hypochondriasis

In the USA, the total cost of hypochondriacal health anxiety and somatisation were 1964.91 US$ per patient for the 12 months preceding and 2089.21 US$ per patient for the 12 months following the index visit. As no information was provided on the financial reference year, we related the cost conversion to the year of data collection (1998) [18]. In Denmark, total costs ranged from 857.19 to 2956.62 US$ per patient per year, depending on the severity of the disease [19, 24]. Risor et al. have examined primary care, secondary care, and indirect costs in terms of productivity loss. The total primary care costs were 452.48 US$, the total secondary care costs were 701.70 US$ and the indirect costs were 1212.65 US$ [24]. Three studies conducted in the United Kingdom reported costs from 1294.95 to 7088.98 US$ per patient per year [21, 25, 26]. The two studies conducted in Sweden were the only ones to examine direct, indirect, and non-medical costs as well. These cost components were also included in the total costs from 15179.29 US$ to 21137.55 US$ per patient per year. According to Hedman et al. the total costs were made up of 4129.14 US$ for direct costs, 1369.27 US$ for non-medical costs and 9680.89 US$ for indirect costs. The indirect costs were therefore more than twice as high as the direct costs [20]. In the study of Axelsson et al. the total costs were made up of 8595.63 US$ for direct costs, 2089.21 US$ for non-medical costs and 10485.87 US$ for indirect costs [17]. The studies regarding the Copenhagen Child Cohort 2000 reflected total costs depending on symptom severity. These ranged between 120.52 and 353.09 US$ per year [22, 23].

### Specific cost components

Five of the included studies examined the medication costs. To enhance comparability, these costs were also converted to annual costs in US$ for the year 2022. In Denmark, this resulted in costs of 400,13 US$ per year regarding DSM-IV diagnosis [19]. In Great Britain, one study showed costs of 2179.70 US$ and the second one 690.06 US$ [21, 26]. Sweden showed a large discrepancy with 39.12 US$ in one publication and 198.96 US$ annual costs in the other [17, 20].

As already mentioned, indirect costs were also examined in 3 publications. These are also made up of various cost components. In all studies, however, the cost item sick leave was examined. This resulted in annual costs of 419.67 US$ and 1432.60 US$ in Sweden and 1212.65 US$ in Denmark [17, 20, 24].

## Discussion

To our knowledge, this is the first systematic review to examine the total costs of health anxiety/hypochondriasis. Our aim was to summarize the available evidence on the costs of hypochondriasis, especially when patients are not receiving treatment, in the form of CBT or other treatment approaches. We were able to identify research gaps and draw implications for future research and policy. This systematic review revealed a lack of data about indirect and direct costs of hypochondriasis. Research on this topic has only been conducted in the United States and 3 different countries across Europe. All of them are high income countries. There were large cost differences observed not only between but also within the countries investigated. The 12-month costs ranged from 857.19 to 21137.55 US$ [17–21, 24–26].

There are several factors that contribute to this heterogeneous data situation. Firstly, the studies did not use standardised definitions and classifications of health anxiety/ hypochondriasis. As previously mentioned the definition of this condition has been subject to major discussions in recent years [6]. In particular, the publication of the latest version of the DSM-V caused major changes, in which the term hypochondriasis was completely removed. However it is retained in the ICD-11 (International Statistical Classification of Diseases and Related Health Problems) [7].

While some studies consider all participants above a certain cut-off point (e.g. short health anxiety inventory score ≥ 18) to be equally affected by health anxiety [18, 20, 21, 25, 26], other studies divide health anxiety into mild and severe. Accordingly, those studies present divided costs, which then turn out to be lower in comparison [19, 22, 23]. Studies that do not distinguish between mild and severe cases may have potentially captured inaccurately high or low costs. This is because we lack information on the number of participants with a severe manifestation, which consequently results in higher costs [17, 20, 21, 24–26]. Another study examined the costs of hypochondria and somatization; therefore, the costs are not directly comparable to the other outcomes as they may be over measured due to the overlap with the second disease component. Furthermore, the data were collected in 1998 and represent the sole existing data from the United States [18]. This highlights the scarcity of data and the subsequent lack of attention given to the condition in this region.

Additionally, different cost items were included in the studies and the type of data collection also influenced the type of cost detail.

This is a widespread issue in health economic studies. A systematic review conducted in 2021 compared various guidelines for health economic evaluations and revealed a substantial overall variability in determining which costs should be included [29].

While especially in Denmark national health registers were used [19, 21–25], the data of other studies were based on hospital databases [18, 26]. Data collection by means of health care registers has the advantage that data is directly available. This means that all costs in the area of primary care can be recorded, regardless of the institution [30]. In particular, the data collection from hospital data could underestimate the costs, as individuals with hypochondriasis tend to engage in “doctor shopping”, which refers to consulting different doctors for the same medical condition. Furthermore the doctor visits outside the hospital are not included in these studies [31].

For the control groups receiving standard care, the knowledge that the participants suffer from hypochondriasis may have influenced the costs. The information about the disease can be viewed as a form of mini-intervention, as the confrontation with one’s own illness may have a therapeutic effect. In such cases, the costs would be lower than expected.

Except for one study, all surveys were conducted in tax-funded health systems with no out-of-pocket payments. This could potentially impact resource utilization and, consequently, costs. However, it can also be inferred that the findings from these studies may not be generalizable to countries with different healthcare systems, particularly low- and middle-income countries.

A systematic review that examined the global economic costs of anxiety disorders has revealed that indirect costs account for a significant proportion of the total costs [32]. Since indirect costs were only investigated in three studies, it is not possible to make a reliable statement about the total costs of hypochondriasis. Although some publications suggest that overall costs are significantly higher when indirect costs are considered, research in this area is still lacking [17, 20, 24].

### Health anxiety/ hypochondriasis in children

Research about hypochondriasis in children appears to be underrepresented. Only two studies, both conducted by the same team within the same cohort, attempted to gain further insights into the economic burden of the disease in this age group [22, 23].

One reason for the missing data could be that diagnostic criteria for hypochondriasis and somatoform disorders for children are still not established. This problem was already identified 25 years ago [28]. The Childhood Illness Attitude Scale was an attempt to identify “characteristics and dimensions of childhood health anxiety” to enable the development of diagnostic criteria for hypochondriasis in children [33]. Interestingly the economic burden resulting from hypochondriasis in children seems to be lower compared to adults. On the one hand, the classification into high and low levels of health anxiety contributes to this phenomenon. On the other hand children are less likely to utilize health services independently because they are dependent on their parents and need to persuade them of the necessity of visiting a doctor [28].

### Limitations

This systematic review is subject to several limitations. Firstly, only articles indexed in English were considered which might have caused a lack of information about cost in non-Englsih speaking countries. Secondly, a limited number of studies were identified for inclusion, and the included studies often contained small study populations. Moreover, the heterogeneity in study design and data collection methods suggests that the results may not be generalizable. Furthermore, it is important to emphasize that the data cannot be generalized, particularly to non-high-income countries due to missing data. The variations in disease definitions and included cost components make data comparability challenging. Lastly, as only total costs were analysed, it is not possible to draw conclusions on specific cost drivers from this research.

### Implications and conclusion

This systematic review offers valuable insights into the costs related to hypochondriasis while identifying relevant research gaps. However, it is important to note that the costs presented in this review are likely underestimated. This is primarily due to the variation in study methodology, where most studies only reported direct costs and employed different definitions of hypochondriasis or health anxiety, including various cost components. Establishing a comprehensive health care system with expanded therapy, prevention, or rehabilitation services relies on solid evidence. For this purpose, a guidance for a standardized and itemized cost survey is essential to identify potential cost drivers that can serve as targets for interventions. Future research especially for policy making should therefore seek to ascertain indirect costs on one hand, as it is likely that these add a significant burden to overall costs. On the other hand, we observed that there is a lack of uniformity of data collection and diagnostic criteria for the condition. Therefore, it is crucial to conduct further studies with consistent data collection and disease definitions.

To be able to assess the costs in a global context, low- and middle-income countries should also be the subject of further research, as only high-income countries have been considered so far. Furthermore, it is important to conduct research that examines the impact of health anxiety on the gross domestic product and the proportion of the total healthcare costs to underline the importance of appropriate interventions and possible savings through them. This could be particularly relevant as an increase in health anxiety in the form of cyberchondria as already explained in the introduction can also be expected in the future due to increasing digitalisation [9].

## Data Availability

All data generated or analysed during this study are included in this published article.

## Abbreviations

A&E: Accident and Emergency department
BIB-CBT: Cognitive Behavioural Bibliotherapy
DSM IV: Diagnostic and Statistical Manual of Mental Disorders
GBP: Great British Pound
G-ICBT: Therapist-Guided Internet Cognitive Behavioural Therapy
CBT: Cognitive Behavioural Therapy
CC: Control Condition
CONSORT: Consolidated Standards of Reporting Trials
HA: Health Anxiety
HAI: Health Anxiety Inventory
iACT: Internet-Delivered Acceptance and Commitment Therapy
iCBT: Internet-Guided Cognitive Behavioural Therapy
iFORUM: Internet Forum
ICD-11: International Statistical Classification of Diseases and Related Health Problems
PRISMA: Preferred Reporting Items for Systematic Reviews and Meta-Analyses
RCT: Randomised Controlled Trial
RCBT: Remotely delivered Cognitive Behaviour Therapy
SCAN: Schedules for Clinical Assessment in Neuropsychiatry
SD: Standard Deviation
SHAI: Short Health Anxiety Inventory
ST: Somatization Threshold
STROBE: The Strengthening the Reporting of Observational Studies in Epidemiology
TAU: Treatment as Usual
U-ICBT: Unguided Internet Cognitive Behavioural Therapy
WLC: Waiting List Control
